# Nursing Staff’s Perspectives of Care Robots for Assisted Living Facilities: Systematic Literature Review

**DOI:** 10.2196/58629

**Published:** 2024-09-16

**Authors:** Katie Trainum, Jiaying Liu, Elliott Hauser, Bo Xie

**Affiliations:** 1 School of Nursing The University of Texas at Austin Austin, TX United States; 2 School of Information The University of Texas at Austin Austin, TX United States

**Keywords:** robots, nursing staff, nursing home, senior living, systematic review, aging, older adults, gerontology, participatory design, user needs, nurses, nursing, retirement, long-term care, geriatrics, elderly, older people, syntheses, review methods, review methodology, searches, searching, systematic, experiences, attitudes, opinions, perceptions, perspectives, preferences, needs, preference

## Abstract

**Background:**

Care robots have been proposed in response to nursing shortages in assisted living facilities (ALFs) and the growing population of older adults. While the use of care robots may improve the general health and well-being of older adults, their introduction changes the work of nursing staff fundamentally, and it has implications for the entire health care system. In developing such technology, it is important to include end users, but so far, the nursing staff’s perspectives have largely been ignored.

**Objective:**

This study aims to examine the literature on nursing staff’s attitudes, needs, and preferences related to the use of care robots in ALFs, in order to discover gaps in the literature and guide future research.

**Methods:**

This review follows the PRISMA (Preferred Reporting Items for Systematic Reviews and Meta-Analyses) 2020 protocol. On May 12, 2023, we searched PubMed, CINAHL Plus with Full Text, PsycINFO, the IEEE Xplore Digital Library, and the ACM Digital Library using predetermined search terms. Included publications, written in English, focused on the predevelopment phase, in which information was gathered on nursing staff’s attitudes, needs, and preferences regarding care robots for ALFs. Publications were excluded if they did not provide peer-reviewed empirical data. The studies’ findings were summarized, coded, and analyzed into major themes using thematic analysis and narrative synthesis. Their quality was assessed using McGill University’s Mixed Methods Appraisal Tool and the Joanna Briggs Institute’s critical appraisal tools.

**Results:**

The final sample included 15 studies. Most of the studies (n=11, 73%) were rated as good quality; however, there was a general lack of reporting on important methodological decisions and sample characteristics. Nursing staff desired care robots that could assist with physically demanding tasks and reduce their workload but had mixed feelings on whether robots could or should assist with social tasks. In addition, nursing staff are concerned about the ethics of care robots, as well as about their safety, accessibility, and operability. The nursing staff’s culture, qualification, and role in the facility may influence their perspectives of care robots. The studies lacked theory-driven designs and large sample sizes. Eight (53%) studies mentioned using a participatory design approach, but a lack of established criteria for what constitutes participatory design leads to varying degrees of methodological quality.

**Conclusions:**

There was consensus among nursing staff that care robots should serve as nursing assistants to reduce workload. Whether robots could or should assist with social tasks remains a question. Further research is needed to mitigate nursing staff’s concerns and understand the socioecological factors that influence their perspectives of care robots and their adoption in ALFs. In addition, theory-driven and large sample size study designs are necessary, as well as work to develop clear criteria for related participatory design research.

## Introduction

Across the world, the population of older adults is growing at an unprecedented rate. It is estimated that the US population of older adults—those aged 65 years and older—will grow from 49 million in 2016 to 95 million in 2060 [1]. This trend in aging will be associated with increased care demands: 70% of older adults will eventually require some form of long-term care, including residential care facilities [2]. Older adults who reside in these facilities rely on nursing staff as their primary caregivers. Depending on the level of care the facility provides, the nursing staff can include advanced practice registered nurses, registered nurses, licensed vocational or practical nurses, certified nursing aids or assistants, patient care technicians or assistants, and unlicensed paid caregivers.

At the same time as care demands rise, assisted living facilities (ALFs), or residential settings that provide long-term care to older adults, face a severe nurse staffing shortage. It is estimated that the number of professional care workers will need to increase by 60% globally (or 13.5 million new care workers) by 2040 just to maintain the current ratio of caregivers to older adults [3]. The COVID-19 pandemic exacerbated this staffing crisis, and the US Bureau of Labor Statistics estimates that an additional 245,600 employees are needed just to return ALFs to prepandemic levels [4]. Nurse burnout has contributed to this shortage and also resulted from it; burnout is associated with increased turnover, high workload, and inadequate staffing [5,6]. In fact, some argue that the staffing crisis is not due to a shortage of available nurses, but to a shortage of nurses willing to work under unsafe conditions [7]. The nurse burnout thus has far-reaching implications for the health of nurses, patients, and health care systems. Burnout is significantly related to poor nurse health; poor quality of care and adverse events for patients; and increased nurse turnover, costing hospital systems US $16,736 per nurse per year employed [6,8,9]. ALFs show higher rates of nurse burnout and turnover than do all other health care settings [4,5].

Care robots, which include both social and assistive robots, show promise for addressing the mismatch between the aging population and the shortage of professional caregivers in ALFs. Interest in care robotics is growing, with a 585% increase in publications on health care robots from 2011 to 2021 [10]. Social robots, which include companion robots, perform work tasks based on “interactional performances” between humans (eg, caregivers and patients) or with pets [11]. Assistive robots (also referred to as mobility or service robots) perform physical work tasks such as lifting patients, helping with activities of daily living, or assisting at mealtimes [11]. Some evidence suggests that care robots may improve the general well-being of older adults [12-14]. In a scoping review of 69 studies, the most commonly reported therapeutic benefits consisted of improved mood and emotional states and increased social interaction [13].

Despite the positive effects for older adults and the frequently stated objective of addressing the nursing shortage, very few studies have managed to reduce workload [15]. Furthermore, the perspectives of nursing staff on the use of care robots have been largely ignored. In the previously mentioned review of 69 studies, only 15 included nursing staff as participants [13]. In those 15 studies, the robots relieved nurses of certain tasks (eg, responding to call lights [16]), but they often required the assistance of staff for operation (27/69, 39%) and thereby increased workload [13,17,18]. A scoping review on the effects of care robots on professional caregivers supports these findings: the introduction of care robots had both positive and negative effects, reducing physical and mental demands in some scenarios and increasing workload in others [11]. These examples highlight the fact that although care robots have the potential to improve the health of older adults, such benefits are in part due to skilled use by nursing staff [19]. Further, the implications of introducing care robots into ALFs extend beyond the recipient of care (ie, older adults) to the entire health care system. Care robots will fundamentally change the nature of nursing work. These findings are important, given today’s climate of widespread nursing shortages and burnout.

Although this research topic is gaining popularity, the usability and acceptability of care robots remain a barrier to widespread adoption. High workloads and negative attitudes have been identified as barriers to staff’s adoption of care robots [20,21]. In their scoping reviews on robots in nursing, Maalouf et al [22] and Ohneberg et al [23] identified usability and acceptance issues, the need for further research on the psychological barriers to acceptance, and the need to improve collaboration between nurses and robots [22,23]. Successful deployment of care robots into health care systems and facilities requires a comprehensive understanding of multiple stakeholders’ perspectives of their use, and their design must be informed by an awareness of the context in which they will be used [24,25].

Participatory design is a research approach that actively involves stakeholders in the design process of emergent technologies to ensure that their needs and preferences are addressed by the developed technology [26-28]. Previous literature reviews have been conducted to examine the participatory design and other similar research approaches for the design and development of care robots for ALFs, but they have mainly included the receivers of care (ie, older adults), leaving the perspectives of nursing staff underexplored [29-31]. In addition, these reviews have aimed to evaluate and compare different research methodologies, not to synthesize participants’ perspectives [29-31]. A 2018 scoping review explored the views of nurses and other health care providers on the use of assistive humanoid and animal-like robots and identified mixed opinions, but more positive than negative, and concerns related to patient safety and privacy [32]. While this review did focus on the perspectives of health care providers, including nurses, the authors excluded robots without a social or interactive element and did not focus on older adults or the ALF setting. Furthermore, this scoping review did not include a quality assessment of the selected articles, which limits its ability to offer suggestions for practice. Our review builds on these findings by including all types of care robots, focusing on the assisted living setting, and including a quality assessment of the reviewed publications. We have chosen to focus on the ALF setting because the world’s growing population of older adults and subsequent rising need for residential long-term care, coupled with widespread staffing shortages, has led to a growing interest in care robots for ALFs [13]. Additionally, it is important robots are customized to the specific end user and health care setting, as the needs of staff and care receivers differ greatly between different care settings [33].

Nurses’ perspectives have not been fully implicated in the design, development, and implementation of care robots. Without consideration of nursing staff and the care environment of ALFs, care robots may further exacerbate the nurse staffing crisis and are unlikely to be adopted into care. To address these research gaps, we have conducted a systematic literature review in order to answer the following research questions: (1) What is known about the attitudes, needs, and preferences of nursing staff in ALFs in relation to the use of care robots? (2) What research methods, designs, and populations have been used in this research? (3) What are the gaps in the literature that warrant future research? The results of this systematic literature review were originally published as part of the 2024 ACM/IEEE (Association for Computing Machinery/ Institute of Electrical and Electronics Engineers) International Conference on Human-Robot Interaction [34]. This study expands on those findings and provides additional methodological details, as well as a quality assessment of the included publications.

## Methods

### Study Design

This literature review follows the PRISMA (Preferred Reporting Items for Systematic Reviews and Meta-Analyses) 2020 protocol for systematic literature reviews [35]. The PRISMA 2020 checklist is provided in Multimedia Appendix 1. A protocol was not registered for this systematic literature review.

### Round 1: Keyword Search

The topic of care robots in ALFs is an interdisciplinary concern; therefore, we searched databases in engineering, computer science, and health sciences: PubMed, CINAHL Plus with Full Text, PsycINFO, the IEEE Xplore Digital Library, and the ACM Digital Library. On May 12, 2023, these databases were searched using the following search terms: (“robot*”) AND (“senior living facilit*” OR “residential facilit*” OR “independent living” OR “assisted living” OR “senior living center*” OR “nursing home*” OR “skilled nursing facilit*” OR “intermediate care facilit*”) AND (“aged” OR “older” OR “elderly”) AND (“nurse*” OR “nursing” OR “staff” OR “professional caregiver*” OR “professional carer*”) AND (“perspective*” OR “preference*” OR “need*” OR “user-centered design” OR “user-driven design” OR “participatory design” OR “co-design” OR “usability” OR “universal design” OR “user experience*”). These search terms build on a previous literature review and were informed by the authors’ previous experience and other relevant literature in the field [13]. To retrieve the full scope of literature on our topic, we imposed no limit for years of publication. PubMed, CINAHL Plus with Full Text, and PsycINFO were searched by titles and abstracts, the IEEE Xplore Digital Library was searched by metadata (titles, abstracts, and indexing terms), and the ACM Digital Library was searched using the 2012 ACM Computing Classification System with the filter “Robotics.” This strategy was adopted from a previous review [13]. A detailed search strategy for each database is provided in Multimedia Appendix 2.

### Round 2: Screening of Titles and Abstracts

Next, the first author screened the retrieved publications by title and abstract using predetermined inclusion and exclusion criteria. To be included, the publications had to meet the following criteria: (1) full text written in English, and (2) focus on the predevelopment phase, on gathering information on nursing staff’s attitudes, needs, and preferences regarding care robots for ALFs. Following a previous literature review [13], we adopted the National Library of Medicine’s MeSH (Medical Subject Headings) definition of robotics: “the application of electronic, computerized control systems to mechanical devices designed to perform human functions” [36]. Smart assistive devices (eg, walkers, canes, and transfer devices) and ambient assisted living technologies without a robotic platform were thus excluded from the review. Studies of the implementation of a care robot or of perspectives of an already developed care robot were also excluded. We defined “assisted living facility” as any residential setting that provides long-term care to older adults, consistent with prior literature reviews [12]. Publications that did not focus on such facilities (eg, aging in place) were excluded. Publications were also excluded if they were not peer-reviewed empirical studies (eg, literature reviews, opinion pieces, system architectures, and dissertations). We included all other research designs: quantitative, qualitative, and mixed methods.

### Round 3: Screening of Full Text

The remaining papers were screened by full text using the same inclusion and exclusion criteria.

### Round 4: Coding and Analysis of Full Text

The final 15 publications for review were coded by publication year, study aim, research method, sample characteristics, country and setting where data collection occurred, and key findings. Qualitative study findings were synthesized using applied thematic analysis to understand the main themes regarding nursing staff’s perspectives of care robots for ALFs [37]. Quantitative data were examined using narrative synthesis to gain a richer understanding of the perspectives of nursing staff, as well as methodological trends and limitations of the existing literature.

In addition, we assessed the levels of evidence reported in the 15 publications. To assess studies with a mixed methods design, McGill University’s Mixed Methods Appraisal Tool was used [38]. For all other study designs, the critical appraisal tools developed by the Joanna Briggs Institute were used to evaluate study quality [39]. To compute scores, questions answered as “Yes” received 1 point and questions answered as “No” or “Unclear” received no points. The total score was then divided by the number of questions and multiplied by 100. Depending on the score, studies were rated as very poor (0%-30%), poor (31%-50%), fair (51%-70%), good (71%-90%), and excellent (>90%). Two reviewers (KT, a doctoral student in Nursing, and JL, a doctoral student in Robotics) independently completed the checklist for each study; their interrater reliability reached 87%; disagreements were resolved through discussion.

## Results

### Search and Screening Results

During round 1, keyword search, the 5 databases yielded 231 publications (n=53, 23% from PubMed; n=44, 19% from PsycINFO; n=42, 18% from CINAHL; n=35, 15% from IEEE; and n=57, 25% from ACM). When these publications were combined, 11 duplicates were identified and removed using Rayyan, an electronic screening tool [40]; a total of 220 nonduplicate publications remained for screening.

**Figure 1:**
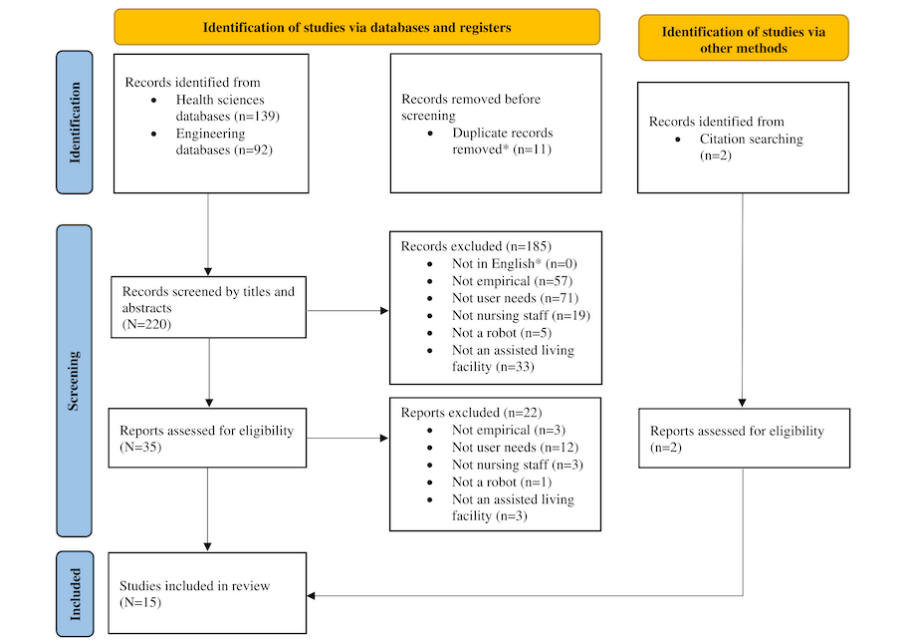
PRISMA (Preferred Reporting Items for Systematic Reviews and Meta-Analyses) flow diagram. *Records that were excluded by automation tools.

During round 2, screening of titles and abstracts, a total of 185 publications were excluded (Figure 1), resulting in 35 publications for full-text review.

In round 3, screening of full text, 22 additional publications were excluded; a total of 13 remained. Additionally, 2 publications were added via citation searches of the included studies, for a total of 15 publications. Figure 1 presents the full search and screening process.

### Quality Assessment Results

Multimedia Appendix 3 [41-55] shows the results of the quality assessment. From the results, 2 (13%) publications were rated excellent, 11 (73%) were rated good, and 2 (13%) were rated fair. The qualitative publications overall lacked philosophical and theoretical frameworks. With regard to the quantitative publications, points were deducted for a lack of valid and reliable instruments, as well as a lack of consideration of confounding variables. Although the majority of studies were rated good, there was a general lack of reporting on important methodological decisions and sample characteristics.

### Descriptive Results Based on the Coding of Full Text

A summary of the 15 studies is presented in Multimedia Appendix 4 [41-55]. The 15 studies in our final sample were published from 2007 to 2023; the majority (8/15, 53%) were published in the last 5 years. They were conducted in 11 countries—in North America (7/15, 47%), Europe (5/15, 33%), Asia (2/15, 13%), and Oceania (2/15, 13%). One study was conducted in both Europe and Asia, so it is accounted for twice [45]. Qualitative designs were most common (8/15, 53%); the remaining studies used mixed methods (3/15, 20%), a cross-sectional approach (3/15, 20%), or quasi-experimental designs (1/15, 6%). Qualitative data collection included interviews (6/15, 40%), focus groups (8/15, 53%), and observation (3/15, 20%). The cross-sectional studies relied on questionnaires. The Robot Anxiety Scale was used in 2 of the 15 (13%) studies; this scale has high reliability (Cronbach α=0.92) [42,43]. Others included A Questionnaire for the Use of a Social Robot in Care for Older Persons [54], which has high reliability (Cronbach α=0.95; intraclass correlation coefficient 0.88) [56], and the Positive and Negative Affect Schedule [42], which has also demonstrated high reliability (Cronbach α=0.84-0.90) and validity [57]. The quasi-experimental study developed new scales from existing literature and calculated high reliability for each scale (Cronbach α=0.70-0.92) [49]. The remaining questionnaires were study-specific and were developed from the literature, from focus groups, or by an undisclosed method, without any reliability or validity testing.

Most of the studies (11/15, 73%) lacked theoretical guidance. Four of the studies included a theoretical framework: the social-ecological model [48], the technology acceptance model [47], the model for the ethical evaluation of sociotechnical arrangements [46], and Forlizzi product ecology [44]. The 4 studies adopted a more holistic approach, exploring the social, environmental, and organizational aspects of ALFs (eg, nursing workflows) in relation to care robots [44,48,52,53]. In addition, 8 studies mentioned using a participatory design or related approach [44,46,48-50,52,53,55]. An overview of how these studies defined and facilitated user participation is presented in Table 1, as well as at which stage of the innovation process the users were involved. Seven studies (47%) used a predetermined definition of “care robot,” meaning that aspects of the robot had been decided before the study took place. Three of these studies focused respectively on robots for a specific task or purpose: robot-assisted feeding systems [48], a robotic shower system [46], and robots to address personal mobility challenges [55]. Five studies involved demonstrations with robotic prototypes [44,49,53-55].

All 15 studies relied on convenience samples. Only 2 focused exclusively on nursing staff [49,52]; the remaining included older adults (10/15, 67%), relatives (4/15, 27%), other staff members (4/15, 27%), or experts in the field (4/15, 27%). Nursing staff included registered nurses (10/15, 67%); certified care workers (eg, certified nursing assistants/aides, licensed vocational nurses, or licensed practical nurses; 3/15, 20%); unspecified caregivers or nursing staff members (10/15, 67%); nurse practitioners (2/15, 13%); and nursing supervisors (2/15, 13%). Participants in 1 study were nursing and medical students [54], and another study included roboticists [55]. Sample sizes ranged from 3 to 286 nursing staff members; a majority (8/15, 53%) had fewer than 10 participants. Four studies provided no additional demographic information besides the participants’ roles in the ALF [41,47,50,55]. Of the studies that did provide additional demographic information, all but one [49] had mainly female participants; the average age of participants ranged from 22.2 to 50 years, and average years of experience ranged from 2.5 to 12 years. Level of education, reported in only 3 studies, ranged from 26% to 43% college educated [43,45,54]. Most studies were conducted in a single setting, but 6 included participants from multiple facilities [42-45,51,55]. Eight of the studies focused on not-for-profit or government-funded facilities [41-43,47,50-53]. Participants were recruited in 1 study through a medical university [54]. Three studies, in addition to investigating ALFs, also examined community-based care for older adults [45,46,55]. Key characteristics of the 15 studies are provided in Table 2.

**Table 1:** Overview of participatory design or related approaches.

Author (year)	Definition/facilitation of user participation	Stage of innovation
Chang and Šabanović (2014) [44]	User-centered design approach: emphasis is on the perspectives of users instead of technology development. Uses task analysis, interviews, field observations, and focus groups.	Predesign
Klein and Schlömer (2018) [46]	User-centered requirements analysis: users are involved in all relevant stages of development. Uses interviews and focus groups to analyze requirements for the shower system, discuss renderings and mock-ups, and identify sociotechnical arrangements and ethical problems.	Predesign; postrenderings and mock-ups
Bhattacharjee et al (2019) [48]	Community-led relational approach shifts away from focusing on a single user to supporting multiple users at multiple layers of the social network. Uses contextual inquiry and surveys.	Postinitial design
Erebak and Turgut (2019) [49]	Human-centered technology approach: human and robot are evaluated as a team to understand the work prior to implementing technology. Participants evaluated existing robots.	Preproduction
Johnson et al (2020) [50]	Need finding design approach prioritizes the needs of older adults first and caregivers and clinicians second. Uses focus groups and surveys.	Predesign
Fiorini et al (2021) [55]	Cocreation or co-design: older adults and caregivers are the principal investigators. Uses interviews.	During design or development
Stegner and Mutlu (2022) [52]	Co-design: to help understand caregivers’ work and guide the design of care robots. Uses observations and interviews.	Predesign
Stegner et al (2023) [53]	Situated participatory design enables the design and testing of use scenarios through interaction with the robot prototype. Uses focus groups.	During design or development

**Table 2:** Key characteristics of the 15 studies in the final sample (N=15).

Characteristic			Frequency, n (%)
**Year of publication**			
	2009 and earlier	2 (13)	
	2010-2014	2 (13)	
	2014-2018	3 (20)	
	2019-2023	8 (53)	
**Country of publication^a^**			
	North America	7 (47)	
	Europe	5 (33)	
	Asia	2 (13)	
	Oceania	2 (13)	
**Sample size**			
	Less than 10	8 (53)	
	11-60	4 (27)	
	61-110	2 (13)	
	111 and greater	1 (6)	
**Method**			
	Qualitative methods	8 (53)	
	Mixed methods	4 (27)	
	Cross-sectional methods	2 (13)	
	Quasi-experimental methods	1 (6)	
**Sample background^a^**			
	Exclusively nursing staff	2 (13)	
	Older adults	10 (67)	
	Relatives	4 (27)	
	Other staff members	4 (27)	
	Content experts	4 (27)	

^a^Studies were accounted for multiple times if met the criteria.

### Nursing Staff Desired Robot Characteristics

Across the 15 studies, nursing staff described their ideal robot as one that could assist with their high workload. The most desired capability of a care robot was to assist with physically demanding tasks (9/15, 60%) [41,43-45,47,48,50,52,55]. These tasks included activities of daily living (eg, bathing, toileting, and feeding) and transferring or lifting patients. The nursing staff also desired a robot that could assist with monitoring patients and alerting staff when patients were in danger (8/15, 53%) [43,45-47,50,52,54,55], physical therapy or mobility exercises (5/15, 33%) [43,45,47,50,54], and medication administration and reminders (3/15, 20%) [45,50,54]. Less commonly desired capabilities included cognitive interventions [47,54], assessments [43], entertainment [44], and assistance with visual or hearing deficits [47].

Although assisting with physical tasks was the most desired function of care robots, participants in 2 studies discussed care that is lost when such work is delegated to robots [46,48]. In one of those studies, the nursing staff discussed how bathing patients involves more than just hygiene—staff used this time to connect with patients and establish relationships, to motivate patients to participate in the bathing process, and to monitor health changes (eg, skin breakdown) [46]. In the other study, staff expressed concerns about the robot replacing important bonding time that occurs during meals [48].

Opinions on whether a care robot could provide social support were mixed. In a few studies, nursing staff believed that care robots were capable of providing such care [51] or a limited version of it [44]. In 1 study, staff had a “wait and see” attitude about robots’ abilities [55]. Although their thoughts about robots’ ability to provide social care were inconclusive, nursing staff agreed that delegating such care to a robot would have serious implications [44-46,51,52,54,55]. In 3 (20%) studies, staff were adamant that robots should not provide social care [46,52,55]. In 3 other studies (20%), staff were less decisive about whether robots should provide social support and feared that their introduction could lead to inhumane or subpar care; however, these participants saw robots as a potential mediator of human social interaction [44,51,54]. For example, participants in 1 study discussed the benefit of using care robots to connect residents with loved ones during the COVID-19 pandemic [51]. In 1 study, beliefs about whether care robots could or should provide social support differed on the basis of the staff’s cultural background [45].

Finally, the nursing staff desired robots personalized to older adults’ abilities, routines, preferences, and needs [46-48,52-54]. To achieve personalized robots, 1 study suggested “end user programming” where nursing staff (ie, the domain experts) could easily customize robots for older adults [52]. This study also suggested that robots should have the ability to learn and adapt from previous experiences [52]. Another study suggested that nursing staff’s intimate relationships with residents could be used to gain knowledge and personalize robots for specific older adults [47].

### Concerns

Nursing staff members voiced several concerns about adopting care robots in ALFs. Ethically, there was disagreement about who should control the robots—the older adults or the nursing staff. In 2 (13%) studies, staff argued that they should supervise and have ultimate control over robots, instead of the older adults [52,54]. Their primary argument for needing control of the robots was to ensure older adults’ safety. In other studies, however, nursing staff were concerned about maintaining the older adults’ autonomy and dignity [46,51,53]. An emergency shutoff button was suggested as a method to increase autonomy and safety [43,44,46-48,51]. In 2 (13%) studies, staff were concerned about surveillance and emphasized the importance of protecting the privacy of older adults and staff [51,54]. Finally, nursing staff worried that robots could replace human caregivers and take away their jobs [42,45,46].

Accessibility was also a common concern across studies. Caregivers in one of the studies thus advocated for “distributive justice,” meaning that everyone should have access to care robots [46]. Care robots can be extremely expensive, but finances (whether those of the older adult or of the facility) should not prevent someone from accessing these devices [46,51]. A staff member in 1 study commented that the high cost of care robots can lead to extreme precautions in order to keep the robots in good working order [51]. As a result, the robots were often locked up when they were needed most (ie, when family or staff were unavailable) [51]. In addition to being financially accessible, robots must be easy to hear and see for those with deficits [43].

Finally, nursing staff were concerned about operability. Nursing staff were concerned that care robots might increase workload, and so they desired robots that would be easy to use [44,51,55], easy to clean and maintain [43,51], flexible [44], and reliable [43]. Three of the studies emphasized that robots should save nursing staff time, which could then be invested into providing relational care [43,46,51]. Also related to operability, 3 (20%) studies pointed to a lack of education and training on care technologies in nursing curricula [43,44,54].

### Potential Factors Influencing Robot Perspectives

In 5 (33%) studies, the authors examined potential factors in participants’ perspectives of care robots—culture, qualification, and the participant’s role in the facility. In 1 study, Finnish staff reported significantly greater fear that robots would cause inhuman care and increase loneliness; whereas, Japanese staff were more likely to believe that robots could reduce anxiety and loneliness [45]. In another study, clinicians (therapists and registered nurses) desired robots to help with medications, safety, and mobility tasks, whereas caregivers (certified nursing assistants and other formal caregivers) desired robots to assist with activities of daily living [50]. Medical students in 1 study were concerned about privacy, whereas nursing students were concerned about robots’ social functions [54]. In 2 studies with residents and nursing staff, the residents felt more positively toward the robots than did the nursing staff [42,43]. Both the residents and nursing staff desired assistance from care robots: residents desired assistance with managing their health (eg, medication reminders), and nursing staff desired job assistance (eg, escorting residents to meals) [42].

## Discussion

### Overview of Findings

In this systematic literature review, we have examined 15 studies of nursing staff’s attitudes, needs, and preferences regarding the use of care robots in ALFs. We have synthesized our findings into three major themes: (1) desired robot characteristics, (2) concerns, and (3) methodological approaches.

### Nursing Staff Desired Robot Characteristics

There was a consensus among nursing staff that care robots should serve as nursing assistants to help reduce high workload; they should not replace nursing work. Assistance with physical tasks was the most desired function. Whether robots could or should assist with social tasks remains a question. However, a previous literature review has identified an overrepresentation of social robots as opposed to assistive robots: 60 of the 69 (87%) included studies in that review investigated social robots [13]. This discrepancy illustrates the necessity of involving stakeholders at the early stages of care robots’ development. Whether robots can provide social assistance is a technical question and will require collaboration with roboticists. Whether robots should provide social assistance is an ethical question that requires the involvement of older adults and their caregivers. Existing participatory research in this field has focused on older adults as the key stakeholders [29-31]; however, it is important that future research involve a wide range of perspectives (including nursing staff) to gain a holistic understanding of care robots for ALFs.

In addition, our findings call for future research on what constitutes nursing work in ALFs. The reviewed studies demonstrate that nursing work is extremely diverse, and much of nursing’s most essential care work goes unseen or is taken for granted, due to gender and power dynamics [58-60]. In a literature review of 121 articles on this topic, the authors describe nursing work as multifaceted and a composite of physical, emotional, cognitive, and organizational labor [60]. Many important yet invisible aspects of care cannot be accounted for by the technical affordances of a robot. In the included study that investigated a robotic showering system, nursing staff suggest that while a robot may be useful for the functional aspects of washing a patient, it may not replace the interpersonal nor observational labor involved in their work [46]. The same is true of the electronic health care record, which has been widely adopted throughout the health care system but reduces nurses’ face-to-face interactions [61]. These examples highlight the unexamined and incomplete notions of what nursing work is today—aspects that developers of such technologies may fail to address [62]. Topol [63] describes a future where medicine harnesses the power of artificial intelligence and robotics to support tasks better suited for automation, freeing nurses and other health care providers to focus on providing real care to patients [63]. To develop ethical, effective, and useful robots, thoughtful discussion and study is necessary on the nature of work that nurses do in ALFs (visible and invisible) and what constitutes good care with robotic systems.

### Concerns

Nursing staff had concerns about the ethics, safety, accessibility, and operability of care robots in ALFs. Opinions about who should have control over the robots were mixed. On the one hand, giving older adults control over a care robot may enhance autonomy, but, on the other, nursing staff are responsible for ensuring residents’ safety. Further research on how to preserve older adults’ autonomy while balancing it with safety is needed. Care robots should be accessible to those who need them, and this should be factored into care robot design, development, and implementation. Accessibility includes financial, physical, and cognitive accessibility. Robots’ operability was especially important to nursing staff, who did not want robots to increase their already high workloads. Future research should consider nurse workflows and how to thoughtfully implement robots in a way that does not increase workload. In addition, in the context of the COVID-19 pandemic and widespread staffing shortages, where nurses already feel undervalued, it is important that robots be introduced as assistants to nurses, rather than replace nursing work.

### Methodological Approaches

Several methodological limitations were identified across the 15 studies.

First, 7 (47%) studies used predetermined definitions of care robots, which limited the implications of their participants’ comments. Participatory design is intended to address the power imbalance between researchers and participants. By involving participants at the very beginning of technology design, stakeholders are empowered to take part in the conceptualization of technologies [27]. When participants act solely as informants, however, study findings are biased by the researchers’ assumptions about end users and the types of robots they would prefer, and participants are limited to thinking only within predetermined definitions, which may lead to incomplete or misleading findings [64]. To fully understand the perspectives of nursing staff at the early stages of robot design, future research should use participatory design to empower nurses to describe their ideal robot without any preconceived ideas. Although a majority of the included studies (8/15, 53%) claimed to take a participatory design or related approach, there is a lack of established criteria for what constitutes participatory design, which leads to varying degrees of participants’ involvement. To increase rigor and strengthen the quality of research in this field, there is a need for future work to develop protocols for participatory design.

Second, the sample sizes were small; 53% (8/15) of the studies had fewer than 10 participants. In addition, the studies provided very limited demographic information on the nursing staff or facilities. This lack of empirical reporting is a common limitation in health informatics research and has been noted in previous literature reviews on care robots [13] and artificial intelligence for caregivers of persons with Alzheimer disease [65]. Only 5 studies included demographic factors in the interpretation of their results, and findings differed across culture, qualification, and role in the facility. To understand the factors that influence attitudes toward and needs or preferences for the use of care robots, it is important to collect and consider demographic information on nursing staff as well as other end-user groups. This can promote the development of care robots that are personalized and useful, as well as increase the likelihood that care robots will be adopted into practice. Six of the studies included multiple facilities but with little exploration of how differences among facilities might influence users’ perspectives of care robots. Studying a single facility may allow detailed exploration, but the inclusion of multiple facilities allows comparisons, with greater potential for generalization. This is especially important in the United States, where licensure and regulations differ greatly between different types of facilities and from state to state.

Finally, several different questionnaires were used to assess nursing staff’s attitudes, needs, and preferences, making it difficult to compare findings across studies, and although some of these instruments have been validated, many have not. Further research is needed to develop and validate relevant instruments for understanding nursing staff’s perspectives of care robots.

### Limitations

The systematic literature review has several limitations. First, only 5 electronic databases were searched, so it is possible that important and relevant studies were missed. To mitigate this possibility, we chose an interdisciplinary selection of databases representing engineering, computer sciences, and health sciences. We also searched the citations of our final sample to reduce the chances of missing pertinent studies and added 2 publications. Second, we included only publications with full text written in English, so it is possible that we missed studies written in other languages. Third, our search terms were not exhaustive, and the language used to describe the participatory design and related research approaches is diverse; therefore, it is possible that we missed important relevant articles. To mitigate this possibility, we reviewed the search terms of previous similar literature reviews. In addition, the language used to describe nursing staff varies, and it is possible that we missed studies that used different terms. Notably, after the conclusion of this review, the authors became aware of a study by Chen et al [66], which investigated the attitudes of Taiwanese health professionals in ALFs toward the use of social robots. The authors validated the Chinese version of attitudes toward the use of social robot questionnaire and found that most participants had positive attitudes toward social robots, these attitudes were positively and significantly correlated with their awareness of robots, and staff working in nursing homes (as opposed to lower-acuity, residential aged care) were significantly more positive toward social robots [66]. These findings support our statement that in order to customize robots to the specific care setting and end user, which in turn will promote the robots’ usefulness and adoption, future research is needed on the different individual- and facility-level factors that influence nursing staff’s attitudes toward care robots.

### Conclusions

In this systematic review of 15 studies on nursing staff’s attitudes, needs, and preferences related to the use of care robots in ALFs, we have found that nursing staff desire care robots that will assist with physically demanding tasks and reduce workload. But the nurses wanted to be able to preserve their interpersonal caring work too. Consideration of ethics, safety, accessibility, and operability informed nurses’ concerns across the studies. Further research is needed to mitigate these concerns and understand the demographic, social, environmental, and organizational factors that influence individuals’ perspectives of care robots and their adoption in ALFs.

## Appendix

Multimedia Appendix 1 PRISMA (Preferred Reporting Items for Systematic Reviews and Meta-Analyses) checklist.

Multimedia Appendix 2 Detailed search strategy.

Multimedia Appendix 3 Quality assessment of the 15 studies in the final sample.

Multimedia Appendix 4 Summary of the 15 studies in the final sample.

## Abbreviations

ACM/IEEE: Association for Computing Machinery/Institute of Electrical and Electronics Engineers
ALF: assisted living facility
MeSH: Medical Subject Headings
PRISMA: Preferred Reporting Items for Systematic Reviews and Meta-Analyses
