# Supplementary material for: Nursing Staff’s Perspectives of Care Robots for Assisted Living Facilities: Systematic Literature Review
Source: JMIR Aging. 2024 Sep 16;7:e58629. doi: 10.2196/58629 (PMC11443223; doi:10.2196/58629)
Supplement: Multimedia Appendix 2 [file aging_v7i1e58629_app2.docx]

The search terms build upon a previous literature review and were informed by the authors’ previous experience and other relevant literature in the field. To retrieve a full scope of the literature on our topic of interest, we imposed no time limit on years of publication. All five of the databases were searched on May 12, 2023.

## PUBMED, CINAHL Plus with Full Text, and PsycINFO:

//Searched by titles and abstracts

1 “robot*”

2 “senior living facilit*” OR “residential facilit*” OR “independent living” OR “assisted living” OR “senior living center*” OR “nursing home*” OR “skilled nursing facilit*” OR “intermediate care facilit*”

3 “aged” OR “older” OR “elderly”

4 “nurse*” OR “nursing” OR “staff” OR “professional caregiver*” OR “professional carer”

5 “perspective*” OR “preference*” OR “need*” OR “user-centered design” OR “user-driven design” OR “participatory design” OR “co-design” OR “usability” OR “universal design” OR “user experience*”

6 1 AND 2 AND 3 AND 4 AND 5

## IEEE Xplore Digital Library:

//Searched by metadata (titles, abstracts, and indexing terms)

“robot*”

AND “senior living facilit*” OR “residential facilit*” OR “independent living” OR “assisted living” OR “senior living center*” OR “nursing home*” OR “skilled nursing facilit*” OR “intermediate care facilit*”

AND “aged” OR “older” OR “elderly”

AND “nurse*” OR “nursing” OR “staff” OR “professional caregiver*” OR “professional carer”

AND “perspective*” OR “preference*” OR “need*” OR “user-centered design” OR “user-driven design” OR “participatory design” OR “co-design” OR “usability” OR “universal design” OR “user experience*”

## ACM Digital Library (ACM Full-Text Collection):

//Searched using the 2012 ACM Computing Classification System’s filter “Robotics”

(“robot*”) AND (“senior living facilit*” OR “residential facilit*” OR “independent living” OR “assisted living” OR “senior living center*” OR “nursing home*” OR “skilled nursing facilit*” OR “intermediate care facilit*”) AND (“aged” OR “older” OR “elderly”) AND (“nurse*” OR “nursing” OR “staff” OR “professional caregiver*” OR “professional carer*”) AND (“perspective*” OR “preference*” OR “need*” OR “user-centered design” OR “user-driven design” OR “participatory design” OR “co-design” OR “usability” OR “universal design” OR “user experience*”).
