# Supplementary material for: Nursing Staff’s Perspectives of Care Robots for Assisted Living Facilities: Systematic Literature Review
Source: JMIR Aging. 2024 Sep 16;7:e58629. doi: 10.2196/58629 (PMC11443223; doi:10.2196/58629)
Supplement: Multimedia Appendix 3 [file aging_v7i1e58629_app3.docx]

Joanna Briggs Institute’s critical appraisal checklist for qualitative studies

| Checklist items | Cohen-Mansfield and Biddison [35], 2007 | Chang and Šabanović [38], 2014 | Klein and Schlömer [40], 2018 | Sefcik et al [41], 2018 | Fiorini et al [49], 2021 | Hung et al [45], 2022 | Stegner and Mutlu [46], 2022 | Stegner et al [47], 2023 |
| --- | --- | --- | --- | --- | --- | --- | --- | --- |
| 1. Is there congruity between the stated philosophical perspective and the research methodology? | 0 | 0 | 0 | 0 | 0 | 0 | 0 | 0 |
| 2. Is there congruity between the research methodology and the research question or objectives? | 1 | 1 | 1 | 1 | 1 | 1 | 1 | 1 |
| 3. Is there congruity between the research methodology and the methods used to collect data? | 1 | 1 | 1 | 1 | 1 | 1 | 1 | 1 |
| 4. Is there congruity between the research methodology and the representation and analysis of data? | 1 | 1 | 1 | 1 | 1 | 1 | 1 | 1 |
| 5. Is there congruity between the research methodology and the interpretation of results? | 1 | 1 | 1 | 1 | 1 | 1 | 1 | 1 |
| 6. Is there a statement locating the researcher culturally or theoretically? | 0 | 0 | 1 | 0 | 0 | 0 | 0 | 0 |
| 7. Is the influence of the researcher on the research, and vice-versa, addressed? | 1 | 0 | 0 | 1 | 1 | 1 | 1 | 1 |
| 8. Are participants, and their voices, adequately represented? | 1 | 1 | 1 | 1 | 1 | 1 | 1 | 1 |
| 9. Is the research ethical according to current criteria or, for recent studies, and is there evidence of ethical approval by an appropriate body? | 1 | 0 | 1 | 1 | 1 | 1 | 1 | 1 |
| 10. Do the conclusions drawn in the research report flow from the analysis, or interpterion, of the data? | 1 | 1 | 1 | 1 | 1 | 1 | 1 | 1 |
| Rating | 80% (good) | 60% (fair) | 80% (good) | 80% (good) | 80% (good) | 80% (good) | 80% (good) | 80% (good) |

Joanna Briggs Institute’s critical appraisal checklist for cross-sectional studies

| Checklist items | Broadbent et al [36], 2009 | Coco et al [39], 2018 | Łukasik et al [48], 2020 |
| --- | --- | --- | --- |
| 1. Were the criteria for inclusion in the sample clearly defined? | 1 | 1 | 1 |
| 2. Were the study subjects and the setting described in detail? | 1 | 1 | 1 |
| 3. Was the exposure measured in a valid and reliable way? | 1 | 1 | 1 |
| 4. Were objective, standard criteria used for measurement of the condition? | 1 | 1 | 1 |
| 5. Were confounding factors identified? | 0 | 1 | 1 |
| 6. Were strategies to deal with confounding factors stated? | 0 | 0 | 0 |
| 7. Were the outcomes measured in a valid and reliable way? | 0 | 0 | 1 |
| 8. Was appropriate statistical analysis used? | 1 | 1 | 1 |
| Rating | 63% (fair) | 75% (good) | 88% (good) |

Joanna Briggs Institute’s critical appraisal checklist for quasi-experimental studies

| Checklist items | Erebak and Turgut [43], 2019 |
| --- | --- |
| 1. Is it clear in the study what is the ‘cause’ and what is the ‘effect’? | 1 |
| 2. Were the participants included in any comparisons similar? | 1 |
| 3. Were the participants included in any comparisons receiving similar treatment/care, other than the exposure or intervention of interest? | 1 |
| 4. Was there a control group? | 0 |
| 5. Were there multiple measurements of the outcome both pre and post the intervention/ exposure? | 0 |
| 6. Was follow up complete and if not, were differences between groups in terms of their follow up adequately described and analyzed? | 1 |
| 7. Were the outcomes of participants included in any comparisons measured in the same way? | 1 |
| 8. Were outcomes measured in a reliable way? | 1 |
| 9. Was appropriate statistical analysis used? | 1 |
| Rating | 78% (good) |

Mixed Methods Appraisal Tool, version 2018

| Checklist items | Broadbent et al [37], 2012 | Bhattacharjee et al [42], 2019 | Johnson et al [44], 2020 |
| --- | --- | --- | --- |
| S1. Are there clear research questions? | 1 | 1 | 1 |
| S2. Do the collected data allow to address the research questions? | 1 | 1 | 1 |
| 1.1. Is the qualitative approach appropriate to answer the research question? | 1 | 1 | 1 |
| 1.2. Are the qualitative data collection methods adequate to address the research question? | 1 | 1 | 1 |
| 1.3. Are the findings adequately derived from the data? | 1 | 1 | 1 |
| 1.4. Is the interpretation of results sufficiently substantiated by data? | 1 | 1 | 1 |
| 1.5. Is there coherence between qualitative data sources, collection, analysis, and interpretation? | 1 | 1 | 1 |
| 4.1. Is the sampling strategy relevant to address the research question? | 1 | 1 | 1 |
| 4.2. Is the sample representative of the target population? | 1 | 1 | 1 |
| 4.3. Are the measurements appropriate? | 1 | 0 | 1 |
| 4.4. Is the risk of nonresponse bias low? | 0 | 1 | 0 |
| 4.5. Is the statistical analysis appropriate to answer the research question? | 1 | 1 | 1 |
| 5.1. Is there an adequate rationale for using a mixed methods design to address the research question? | 0 | 1 | 1 |
| 5.2. Are the different components of the study effectively integrated to answer the research question? | 0 | 1 | 1 |
| 5.3. Are the outputs of the integration of qualitative and quantitative components adequately interpreted? | 0 | 1 | 1 |
| 5.4. Are the divergences and inconsistencies between quantitative and qualitative results adequately addressed? | 0 | 1 | 1 |
| 5.5. Do the different components of the study adhere to the quality criteria of each tradition of the methods involved? | 1 | 1 | 1 |
| Rating | 71% (good) | 94% (excellent) | 94% (excellent) |
