# Supplementary material for: Nursing Staff’s Perspectives of Care Robots for Assisted Living Facilities: Systematic Literature Review
Source: JMIR Aging. 2024 Sep 16;7:e58629. doi: 10.2196/58629 (PMC11443223; doi:10.2196/58629)
Supplement: Multimedia Appendix 4 [file aging_v7i1e58629_app4.docx]

Multimedia Appendix 4

Summary of the 15 Studies in the Final Sample

| **Author** | **Year** | **Country** | **Study Aim** | **Method** | **Nursing Staff Participants** | **Sample Demographics** | **Non-Nursing Participants** | | **Setting** | | **Key Findings** | |  |
| --- | --- | --- | --- | --- | --- | --- | --- | --- | --- | --- | --- | --- | --- |
| Cohen-Mansfield and Biddison [35] | 2007 | USA | To determine what technologies the staff were currently familiar with and what technologies they desire | Qualitative (focus group) | 1 charge nurse, 4 CNAs | n/a | Older adults | | 562 bed, non-profit skilled nursing facility (dementia unit) | | Staff universally welcomed technology to help with their job, especially with the most physically demanding tasks (e.g., bathing, toileting, transferring) | |  |
| Broadbent et al [36] | 2009 | New Zealand | To examine staff’s attitudes towards and preferences for healthcare robots | Cross-sectional | 30 staff members (13% RNs, 20% caregivers, 26% managerial, 40% other) | Mean age: 50.17; 70% female | Older adults | | Selwyn Village (600 bed, non-profit community care and ALF) | | Residents were significantly more positive about robots than staff; residents prioritized healthcare tasks vs. caregivers prioritized assistance with their jobs | |  |
| Broadbent et al [37] | 2012 | New Zealand | To study staff’s attitudes towards and preferences for robots | Mixed methods (survey, focus group) | Survey – 4 RNs, 6 caregivers, 8 managerial staff, 12 other  Focus groups – 6 managers, 8 caregivers | Survey – mean age: 50; 70% female; 43% college degree | Older adults, relatives | | Selwyn Village (600 bed, non-profit community care and ALF) | | Staff lacked prior knowledge of care robots; staff can see the potential of robots for lifting, activities of daily living, mobility, and medical assessments (will free staff to spend more time with residents); believe robots could enhance residents’ autonomy; robots are potentially better than humans at monitoring residents (and alerting staff when help is needed); needs to be easy to see and hear; needs to be easy to clean; concerns about residents’ safety, robots’ reliability, and job security; residents have significantly better attitudes toward robots than staff | |  |
| Chang and Šabanović [38] | 2014 | Taiwan | To examine what type of robot design ALF staff find acceptable; gain understanding of the context of ALFs, human-technology relations, technology adoption, and technology perceptions | Qualitative (observation, focus group, interview) | 2 managers, 3 RNs, 2 staff members, 1 RN supervisor, 2 caregivers | RN – Taiwanese  Caregivers – immigrants | Relatives | | 2 rural nursing institutions | | Staff has minimal technology experience and prefers non-digital; concerns about technology adoption because past experiences exacerbated their busy routines; desire a robot that is easy to use and flexible, assistance with minor tasks (e.g., responding to resident requests) and entertaining older adults; robots should have a level of human-like caring, but should not replace complex human tasks; desire help with more demanding tasks (e.g., showering), but have little confidence in robots’ capabilities | |  |
| Coco et al [39] | 2018 | Finland & Japan | To examine and compare staff’s attitudes toward care robots in Finland and Japan | Cross-sectional | Finland – 200 (71% LVN)  Japan – 86 (80% certified care workers) | Finland – mean age: 43; 94% female; mean years of experience: 12  Japan – mean age: 41; 65% female; mean years of experience: 12 | Social workers | | Finland – home care facilities  Japan – 3 residential care homes & 2 rehabilitation day centers | | Japanese scored robots more useful than Finnish; Japanese significantly more likely than Finnish to report robots can help with household tasks, activities of daily living, easing anxiety and loneliness; Finnish significantly more likely than Japanese to report robots can help with medication reminders and physical exercise; Finnish were significantly more fearful of robots leading to inhuman care and increased loneliness than Japanese | |  |
| Klein and Schlömer [40] | 2018 | Germany | To identify the most important ethical issues surrounding a robotic showering system | Qualitative (interview, focus group) | Interview – 15 professional caregivers  Focus groups – 9 professional caregivers | Age range: 36 – 44; 56% female | Older adults | | Nursing care home & model flat | | Shower time is more than just hygiene – staff use this time for building relationships, promoting mobility and autonomy, and monitoring health changes; safety is a priority (recommend fall alarm and emergency stop); emphasized distributive justice; worried about job security and nature of future work; residents and staff agree that robots should not replace human relational work and instead robots should save staff time for essential care tasks; staff desires control of the robots | |  |
| Sefcik et al [41] | 2018 | USA | To discover older adults’ needs to guide the design of a low-cost mobile robot | Qualitative (focus group) | 6 caregivers, 1 NP, 2 RNs | n/a | Older adults, physicians, physical therapists, occupational therapists | | Low-resource, supportive apartment living | | All stakeholder groups desire robot to assist with activities of daily living, transferring, emergency responses (e.g., falls), communication challenges related to hearing deficits, and mobility; ensuring safety is a priority; use staff’s personal connections with residents to personalize robots; robots should be used to increase socialization | |  |
| Bhattacharjee et al [42] | 2019 | USA | To gain understanding on how social relationships influence design and tasks for a robot-assisted feeding system | Mixed methods (survey, interview, observation, focus group) | 5 caregivers (RNs, NPs) | 60% female | Older adults, experts | | Assisted living community | | Caregivers find feeding tasks physically challenging; safety is important, caregivers suggest an emergency stop button; robot should adapt to changing environment; robot should promote safety, privacy, and security; robot should be customizable to user’s preferences; value of robot for enhancing independence, but feeding is an important time for caregiver companionship | |  |
| Erebak and Turgut [43] | 2019 | Turkey | To examine the relationships between trust, anthropomorphism, intention to work with, and preferences for automation level in a robot | Quasi-experimental | 102 caregivers | Mean age: 31; 58% male; mean years of experience: 5; 33% college degree | n/a | | Nursing home | | Robots’ level of anthropomorphism does not impact trust; trust of robot is significantly related to intention to work and preference of automation levels | |  |
| Johnson et al [44] | 2020 | USA | To identify and prioritize the tasks an affordable service robot should be able to accomplish | Mixed methods (survey, focus group) | Focus group – 8 clinicians (therapists, RNs), 6 caregivers (CNAs, home health assistants)  Survey – 14 clinicians, 15 caregivers | n/a | Older adults, therapists | | Federally funded program including ALF settings | | Clinicians’ highest priority tasks were helping older adults with medications, home safety, and transfer/walking vs. caregivers’ highest priority tasks were assistance with care plans, older adults’ physical/mental health challenges, bathing, and feeding; older adults prioritized assistance with everyday tasks vs. clinicians prioritized health/safety; suggest a low-cost robot that accomplishes everyday tasks (e.g., ambulation, feeding), increases social connections, and supports clinical interventions/safety via education, monitoring, and reminders | |  |
| Łukasik et al [48] | 2020 | Poland | To understand future healthcare professionals’ opinions about assistive robots for older adult care | Cross-sectional | 110 nursing students | Mean age: 22.2; completed 2^nd^ year of study; no prior robot experience; high technological literacy; 13% had experience caring for family member | | Medical students | | Medical university | | Robots should provide medication reminders, ensure safety, monitor health status/environment, cognitive training, physical activity motivation, be personalized, and caregiver should have control over robot (not older adult); robot should be a companion/assistant (not replace human relationships); majority felt robot was useful, but nursing students were more positive than medical students; nursing students more focused on social functions vs. medical students focused on privacy issues | |
| Fiorini et al [49] | 2021 | Italy & Netherlands | To identify and prioritize older adults’ personal mobility needs, examine attitudes towards assistive robots, and present technical requirements | Qualitative (interview) | Italy – 3 RNs  Netherlands – 4 RNs | n/a | | Geriatricians, informal caregivers, older adults, roboticist | | Italy – geriatrics unit  Netherlands – long-term care organization | | Caregivers and older adults desire assistance with mobility, rehabilitation, safety, and activities of indoor daily life; caregivers also desire assistance with physically demanding work tasks to prevent injury and monitoring; majority had positive attitudes, but “wait and see” attitude (believe technology is currently too limited to help); worried robots will reduce human social contact; important for robots to be easy to use | |
| Hung et al [45] | 2022 | Canada | To examine the perceived risks and ethical concerns related to the adoption of robots | Qualitative (interview) | 3 nurses, 3 care workers | 80% female; “diverse in ethnic backgrounds”; new and experienced | | Rehabilitative staff, recreation staff, residents, relatives | | 2 long-term care homes | | Safety concerns due to users’ cognitive/physical disabilities or robot malfunctioning; concerned about robot increasing workload due to maintenance and training; privacy, surveillance, and consent concerns (important to have freedom to decline); social justice concerns related to cost; concerns about replacing human connection, but robots can help with loneliness (balance is key); most positive about robots’ potential to meet older adults’ social and emotional needs | |
| Stegner and Mutlu [46] | 2022 | USA | To examine caregiver workflows and practices to determine how robots can assist | Qualitative (observation, interview) | 7 caregivers | Mean age: 50; 100% female; mean years of experience: 11.8 | | n/a | | Suburban, private, not-for-profit senior living facility | | Robots should have multiple capabilities and caregivers should have control over the robot; robots should be customizable and learn/adapt from past experiences; robots should be aware and respond to residents’ mental states (but not provide social support); important to maintain residents’ autonomy | |
| Stegner et al [47] | 2023 | USA | Engage older adults to design an assistive mobile robot and gain understanding of the real-life environments, interactions, activities, and caregiving practices of older adults that impact technology integration | Qualitative (focus group) | 3 caregivers | Mean age: 33.3; 100% female; mean years of experience: 2.5 | | Older adults | | Suburban, private, not-for-profit senior living facility | | Importance of personalizing robot; familiarity with the robot helped shape caregivers’ expectations for what the robot can/should do; caregivers and older adults should share control of robot (balancing safety and autonomy) | |

Note: CNA, certified nursing assistant/aide; RN, registered nurse; ALF, assisted living facility; LVN, licensed vocational nurse; NP, nurse practitioner
